# Effects of Plyometric Training on Lower Body Muscle Architecture, Tendon Structure, Stiffness and Physical Performance: A Systematic Review and Meta-analysis

**DOI:** 10.1186/s40798-022-00431-0

**Published:** 2022-03-21

**Authors:** María Ramírez-delaCruz, Alfredo Bravo-Sánchez, Paula Esteban-García, Fernando Jiménez, Javier Abián-Vicén

**Affiliations:** Performance and Sport Rehabilitation Laboratory, Faculty of Sports Sciences, University of Castilla-La Mancha, Avda. Carlos III S/N, 45071 Toledo, Spain

**Keywords:** Jump training, Physical activity, Myotendinous adaptations, Mechanical properties, Strength

## Abstract

**Background:**

Plyometric training (PT) has been widely studied in sport science. However, there is no review that determines the impact of PT on the structural variables and mechanical properties of the lower limbs and physical performance.

**Objective:**

The aim of this systematic review and meta-analysis was to determine the effects of PT on lower body muscle architecture, tendon structure, stiffness and physical performance.

**Methods:**

Five electronic databases were analysed. The inclusion criteria were: (1) Availability in English; (2) Experimental studies that included a PT of at least eight sessions; and (3) Healthy adults subjects. Four meta-analyses were performed using Review Manager software: (1) muscle architecture; (2) tendon structure; (3) muscle and tendon stiffness; (4) physical performance.

**Results:**

From 1008 search records, 32 studies were eligible for meta-analysis. Muscle architecture meta-analysis found a moderate effect of PT on muscle thickness (Standard Mean Difference (SMD): 0.59; [95% Confidence Interval (CI) 0.47, 0.71]) and fascicle length (SMD: 0.51; [95% CI 0.26, 0.76]), and a small effect of PT on pennation angle (SMD: 0.29; [95% CI 0.02, 0.57]). The meta-analysis found a moderate effect of PT on tendon stiffness (SMD: 0.55; [95% CI 0.28, 0.82]). The lower body physical performance meta-analysis found a moderate effect of PT on jumping (SMD: 0.61; [95% CI 0.47, 0.74]) and strength (SMD: 0.57; [95% CI 0.42, 0.73]).

**Conclusion:**

PT increased the thickness, pennation angle and fascicle length of the evaluated muscles. In addition, plyometrics is an effective tool for increasing tendon stiffness and improving jump and strength performance of the lower body.

## Key Points

- Plyometric training is an effective tool to increase *muscle thickness* of the vastus lateralis, vastus medialis, rectus femoris and triceps surae.
- Plyometric training is effective in increasing *fascicle length* of the vastus lateralis and rectus femoris muscles, and *pennation angle* of the rectus femoris muscle.
- Plyometric training is considered an effective tool for increasing *tendon stiffness*.
- Plyometric training produces improvements in *jump performance* (CMJ, SJ, DJ) and lower body *strength performance.*
- *Muscle and tendon CSA*, *muscle stiffness* and *sprint performance* show no significant changes after a plyometric training programme.

## Introduction

*Plyometric training* (PT) is a type of strength training widely used in team and individual sports to improve sport-specific performance [1, 2]. Plyometric exercises have been shown to be an effective method of improving a number of physical qualities such as strength and jump height [3], running economy [4], agility [5], sprint speed and endurance [6]. The exercises involved in PT are characterised by explosive muscle extension and contraction [1]. These specific exercises consist of three phases: (1) the pre-activation phase (eccentric phase); (2) the amortisation phase (isometric phase); and (3) the shortening phase (concentric phase) [1]. The quick transition from the eccentric to the concentric phase of the movement is known as the stretch–shortening cycle (SSC) [7]. In the eccentric pre-activation phase of plyometrics, the Golgi tendon organs are stretched more than in regular strength training which leads to a greater inhibition of their protective function and leads to an increase in concentric power output [1, 8]. Thus, PT can improve the mechanical characteristics of the muscle–tendon complex, strengthen the elastic properties of connective tissue and optimise cross-bridge mechanics and motor unit activation [7, 9]. These adaptations are associated with improvements in muscle strength, dynamic stability and neuromuscular control, as well as with an increase in contraction speed and joint stiffness [7, 8]. In addition, the recent literature has demonstrated the efficacy of PT in different health-related contexts [10]. Therefore, PT is an effective type of training to improve both *physical performance* [11] and health [10] in athletic and non-athletic populations.

Skeletal *muscle architecture* is usually defined by fascicle length, cross-sectional area (CSA), muscle thickness and pennation angle [12]. These parameters show information about muscle function and are usually employed for musculoskeletal models [13]. It has been suggested that an increase in muscle CSA is accompanied by improvements in force production and larger muscle fibre pennation angles which may increase the number of cross-bridge interactions [14]. Despite PT resulting in a wide range of different physiological and biomechanical adaptations [7, 15, 16], changes in muscle architecture have been less studied [17, 18]. In one of the latest reviews on plyometrics, two types of training (plyometrics vs. resistance) were compared and both were shown to have similar effects on lower limb muscle hypertrophy [19]. Therefore, it is necessary to analyse the results of studies in which a PT programme has been carried out and its effect on muscle architecture has been studied.

*Tendon structure* commonly described as tendon CSA, tendon length or tendon thickness, serves the function of holding the muscle to the bone. Tendons are located at each end of the muscle being firmly connected to the muscle fibres and to the components of the bone [20]. The main function of tendons is to store and transmit the mechanical force of muscle contraction to the bones [21]. High tendon CSA values as a result of adaptation to the type of training should allow the individual to withstand greater mechanical stress [22] and reduce the risk of injury [23]. In fact, 80% of Achilles tendon ruptures occur in the proximal area to the calcaneal insertion, where the tendon is narrowest and has the lowest CSA of the entire structure [24]. There is controversy about the PT effects on tendon structural properties. Houghton et al. [25] and Paleckis et al. [26] found that the Achilles tendon CSA increased after a PT programme. However, other research did not find changes in tendon CSA and tendon thickness after PT [26–28]. We found no studies evaluating the effects of several weeks of PT on tendon length or tendon thickness. As adaptation of the tendon to the rapid eccentric forces may reduce their detrimental effect [29], it is necessary to clarify the effects of PT on tendon structural properties. In addition, eccentric exercises can also be investigated for their possible use as a preventive measure in addition to their rehabilitative role [30].

*Stiffness* is the biomechanical property of the tissue that explains its resistance to a contraction or to an external force that deforms its initial shape [31]. The mechanical properties of tendons have been related to dynamic performance, showing that high stiffness values are beneficial for both rapid SSC activities, as well as for actions involving high speed of movement [32]. Therefore, the rapidity of plyometric exercises, which involve a rapid stretching of the muscle–tendon complex followed immediately by muscle shortening [7], could improve the force transmission to the bone [33, 34]. In PT, the stored energy in the muscle–tendon complex during the stretching phase is used during the shortening phase and transformed into movement without being wasted in the form of heat [1]. The mechanical properties of the muscle–tendon complex have been shown to change after PT [28, 35]. However, the effects of PT on stiffness are unclear, as some studies have found no significant changes in tendon stiffness after PT [28, 36], while other studies found significant increases showing improvements in force transmission to bone [37–39]. Therefore, a thorough evaluation is needed to discuss the nature of the possible physiological mechanisms involved in the changes in mechanical properties after PT [38] and to find out what differences exist among studies so that the changes in stiffness after PT are not the same.

The systematic reviews and meta-analyses that can be found to date on the effects of PT base their research mainly on physical performance parameters, and few results are found on the effects on muscle architecture, tendon structure and stiffness. Therefore, the aim of this systematic review and meta-analysis was to determine the effects of PT on lower body muscle architecture, tendon structure, stiffness and physical performance.

## Methods

### Study Design and Registration

PubMed, Scopus, Web of Science (WOS), MEDLINE and SportDiscus databases were systematically searched for articles describing the effects of PT.

This systematic review followed the Preferred Reporting Items for Systematic reviews and Meta-Analyses (PRISMA) [40]. The International Prospective Register of Systematic Reviews (PROSPERO) registration number is (CRD42020219228).

### Search Strategy and Study Selection

A manual search was performed using a combination of the following key terms: plyometric training, muscular architecture and tendon structure. These concepts were applied using the search operator “AND” in title and abstract. The full search string is provided in Appendix A.

The databases were searched for articles published up to 25 January 2022. After removing the duplicated studies, two researchers (M.R.C. and A.B.S.) independently screened titles and abstracts to identify articles meeting the inclusion criteria described below. If the two assessors did not agree about article selection, consensus was sought in a meeting. If necessary, a third author (P.E.G.) was consulted to make the final decision.

The full text of the selected articles was retrieved and independently screened by the same researchers to determine whether articles met the inclusion criteria. The reference lists of the included articles were checked to ensure no publications were missed by the initial search and authors were contacted for missing outcomes if necessary.

### Eligibility Criteria

To be included in the present systematic review, studies had to satisfy the following inclusion criteria: (1) Availability in English; (2) Experimental studies that included a PT programme of at least eight sessions, to determine the effects on lower limb (pre- and post-training); (3) Carried out on adult men and/or women (≥ 18 years) without pathologies or health problems.

We excluded articles that (1) were review articles, editorials or letters to the editor or case reports; (2) were performed on animals, cadavers or in vitro; (3) did not provide data on post-training; (4) were observational studies that did not apply any type of PT.

### Methodological Quality Assessment

Quality assessment was carried out by two authors (M.R.C. and A.B.S.) using The Physiotherapy Evidence Database (PEDro) scale [41]. Quality assessment was completed before data extraction was started. The PEDro scale consists of 11 items designed to assess the methodological quality of the studies. Each satisfied item contributes 1 point to the overall PEDro score (range 0–10 points). Item 1 was not included as part of the study quality rating as it pertains to external validity. Thus, quality assessment was interpreted using the following 10-point scale: 0–3 points were considered poor quality, 4–5 points as moderate quality and 6–10 points as high quality [42]. The table with PEDro scale is provided in Appendix B.

The two same authors independently performed risk of bias assessment for the included studies. Cochrane Robins 2.0 for randomised trials was used [43]. This tool assesses methodological quality and indicates potential risk of bias on the basis of 7 aspects: (1) random sequence generation; (2) allocation concealment; (3) blinding of participants and personnel; (4) blinding of outcome assessment; (5) incomplete outcome data; (6) selective reporting; and (7) other bias. The overall judgement was summarised as “low risk of bias”, “some concerns” or “high risk of bias”. The Kappa correlation test was used to analyse the level of agreement among authors in order to control for risk of bias of the included studies. The level of agreement obtained was *k* = 0.88. The details of the risk of bias assessment of the included trials are displayed in Appendix C. In case of disagreement between the two assessors about quality assessment or risk of bias assessment, consensus was sought in a meeting. If necessary, the third author (J.A.V.) was consulted to make the final decision.

### Data Extraction

The full texts of each study were collected, and the necessary data were extracted from both the text and tables. The data extraction was performed independently by two reviewers (M.R.C. and A.B.S.), and a third author (J.A.V.) was consulted to resolve disagreements where necessary. Data were compiled in a document produced using a standardised data extraction programme. In the absence of essential data in the original studies, authors were contacted for the necessary information. The data extracted were: (1) name of the first author and year of publication; (2) characteristics of the population, with the total sample and by groups, age and physical fitness of the participants; (3) characteristics of the PT programme, where the duration in weeks, number of training days per week, total number of sessions, minutes per session and total jumps performed were collected; and (4) selected variables, in turn divided into results referring to lower body muscle architecture, tendon structure, stiffness and physical performance (Table 1).

**Table 1:** Summary of included studies

Study	Population			Characteristics plyometric training programme	Selected Variables					
	Sample size (N): Male (M)/Female(F)	Age (years)	Physical fitness		Muscle architecture	Tendon structure	Muscle and tendon stiffness	Lower body physical performance		
								Jump	Sprint	Strength
Blazevich et al. [49]	*N* = 23 (15M/8F) SQ = 8 (5M/3F) FHS = 7 (5M/2F) SJT = 8 (5M/3F)	22.1 ± 1.9	Competitive athletes such as rugby union, rugby league, soccer, or netball players	9 weeks 4 days/week 36 sessions 60 min/session	VL Muscle thickness RF Muscle thickness VL Fascicle angle RF Fascicle angle VL Fascicle length RF Fascicle length			Force and displacement in CMJ	20 m sprint time	Forward hack squat double leg
Burgess et al. [37]	*N* = 13 (M) PT = 7 IT = 6	23 ± 6	Not defined	6 weeks 2–3 days/week 950 jumps approx			Medial gastrocnemius tendon stiffness	SJ height		Concentric rate of force development
Coratella, et al. [50]	*N* = 48 (M) BMSJT = 16 WJST = 16 CG = 16	21 ± 3	Recreational soccer players	8 weeks 2 days/week 16 sessions 20–25 min/sessions BMSJT = 800 jumps WJST = 656 jumps	VL Muscle thickness VL Pennation angle VL Fascicle length			CMJ height SJ height	30 m sprint time	Quadriceps concentric peak-torque
Correa et al. [60]	*N* = 58 (F) EG = 41 After 6 weeks was further divided into 3 subgroups (TG = 14, PG = 13, RG = 14) CG = 17	67 ± 5	Older women	12 weeks (divided into two 6-week phases) 2 days/week 24 sessions	VL Muscle thickness VM Muscle thickness RF Muscle thickness			CMJ height		1 RM-knee extension
Fouré et al. [36]	N = 17 (M) PT = 9 CG = 8	PT 18.8 ± 0.9 CG 18.9 ± 1.0	Explosive sport practice (basketball, volleyball and handball)	8 weeks 2 days/week 16 sessions 60 min/session 3200 jumps			Maximal stiffness of gastrocnemius Achilles tendon stiffness	SJ height		Maximal torque in plantar flexion
Fouré, et al. [38]	*N* = 19 (M) PT = 9 CG = 10	PT 18.8 ± 0.9 CG 18.9 ± 1.0	Regular sport practice	14 weeks 2–3 days/week 34 sessions 60 min/session 6800 jumps		Achilles tendon CSA	Tendon stiffness	CMJ height SJ height		MVC
Fouré et al. [18]	*N* = 19 (M) PT = 9 CG = 10	PT 18.8 ± 0.9 CG 18.9 ± 1.0	Regular sport practice	14 weeks 2–3 days/week 34 sessions 60 min/session 6800 jumps	CSA Triceps surae Pennation angle triceps surae (medial gastrocnemius. lateral gastrocnemius, soleus) Fascicle length triceps surae (medial gastrocnemius. lateral gastrocnemius, soleus)	Achilles tendon CSA	Passive part of the series elastic component stiffness (gastrocnemius tendon)	CMJ height SJ height		
Fouré et al. [59]	N = 19 (M) PT = 9 CG = 10	PT 19.6 ± 1.8 CG 22.1 ± 3.7	Regular sport practice	14 weeks 2–3 days/week 34 sessions 60 min/session 6800 jumps	CSA of the gastrocnemius muscles	Achilles tendon CSA	Stiffness index of Achilles tendon Stiffness index of gastrocnemius muscles			Maximal passive force of gastrocnemius
Franchi et al. [51]	*N* = 23 (M) YM = 14 OM = 9	YM 25.4 ± 3.5 OM 69.7 ± 3.4	Young and older men	6 weeks 3 days/week 18 sessions	VL Muscle thickness VL Pennation angle VL Fascicle length					MVC
Grosset, et al. [65]	*N* = 30 (20 M/10F) PT = 9 (6 M/3F) ET = 21 (14 M/7F)	PT 21.0 ± 2.3 ET 21.3 ± 0.3	Sedentary college students	10 weeks 2 days/week 20 sessions 25–45 min/session 5000 jumps				DJ height		MVC
Helland et al. [52]	*N* = 39 (29 M/10F) OWL = 13 (9 M/4F) MSPT = 13 (10 M/3F) FSPT = 13 (10 M/3F)	20 ± 3	Badminton, volleyball, and hockey players from a Norwegian High School for elite sports	8 weeks 2–3 days/week 21 sessions OWL 45 min/session MSP 25 min/session FSP 35 min/session	VL Muscle thickness RF Muscle thickness VL Pennation angle VL Fascicle length			CMJ height SJ height DJ 40 cm height	30 m sprint time	1 RM squat
Hirayama et al. [63]	*N* = 21 (M) PT = 11 CG = 10	PT 22 ± 3 CG 22 ± 4	Recreationally active males	12 weeks 3 days/week 36 sessions			Achilles tendon stiffness			Static plantar flexion torque
Horiuchi et al. [66]	*N* = 20 (M) BFR = 10 CG = 10	BFR 23.3 ± 2.5 CG 22.4 ± 1.7	Untrained healthy males	4 weeks 4 days/week 16 sessions				CMJ height SJ height		Knee extension
Horwath et al. [53]	*N* = 22 (M) ISO/ECC = 11 TRAD = 11	18 ± 1	Resistance-trained ice hockey players	8 weeks 2–3 days/week 21 sessions	VL Muscle thickness VM Muscle thickness RF Muscle thickness			CMJ height DJ height	30 m sprint time	1 RM
Houghton et al. [25]	*N* = 15 (M) PT = 7 CG = 8	21.6 ± 4	Players from local district-grade cricket clubs	8 weeks 2–3 days/week 16–24 sessions 1785 jumps		Achilles tendon CSA	Achilles tendon stiffness	CMJ height SJ height	5 m sprint time	
Hunter and Marshall [67]	*N* = 50 (M) *P* = 11 *S* = 11 PS = 14 CG = 14	24 ± 4	Athletes (primarily basketball and volleyball players), with little experience in endurance training	10 weeks 2 days/week 20 sessions 25–60 min/session				CMJ height DJ 30 cm height		
Kijowksi et al. [68]	*N* = 19 (M) TG = 9 CG = 10	TG 21.1 ± 1.2 CG 22.5 ± 2.0	Subjects had at least 2 years of previous experience with recreational strength and power training and were not involved in any competitive athletic sports	4 weeks 2 days/week 8 sessions						Squat 1RM/BM
Kubo et al. [28]	*N* = 10 (M) PT = 10 WT = 10 Subjects performed plyometric training on one side (PT) and weight training on the other side (WT). In each subject, the right and left legs were randomly allocated to the training protocols	22 ± 2	Healthy males without experience of regular exercise training	12 weeks 4 days/week 48 sessions		Achilles tendon CSA	Achilles tendon stiffness	CMJ height SJ height DJ height		MVC
Kubo et al. [54]	*N* = 11 (M) PT = 11 IT = 11 One leg performed plyometric training (PLY) and the other leg performed isometric training (ISO). In each subject, the right and left legs were allocated to the training protocols in a random manner	22.5 ± 3.2	Healthy males without experience of regular exercise training	12 weeks 3 days/week 36 sessions	Muscle thickness of triceps surae (medial gastrocnemius. lateral gastrocnemius, soleus)	Achilles tendon CSA	Stiffness Passive muscle stiffness	CMJ height DJ height		MVC
Kubo et al. [61]	*N* = 21 (M) PT = 11 CG = 10	PT 20.9 ± 1.6 CG 21.0 ± 1.4	Healthy male college students (any regular training for at least 2 years before)	12 weeks 3 days/week 36 sessions	Muscle thickness of triceps surae (medial gastrocnemius, lateral gastrocnemius, soleus)	Achilles tendon CSA	Achilles tendon stiffness	CMJ height SJ height DJ height		
Kudo et al. [55]	*N* = 21 (14 M/7F) ECR = 11 (8 M/3F) NCR = 10 (6 M/4F)	ECR 21.2 ± 3.9 NCR 19.6 ± 0.7	Healthy and normal volunteers	12 weeks 7 days/week 84 sessions ECR 15 × 3 repetitions daily in two sets NCR 20 × 3 repetitions daily in two sets	Muscle thickness of triceps surae (medial gastrocnemius) Pennation angle of triceps surae (medial gastrocnemius) Fascicle length of triceps surae (medial gastrocnemius)					
Laurent et al. [27]	*N* = 32 (17 M/15F) TG KE = 11 (6 M/5F) TG KF = 11 (6 M/5F) CG = 10 (5 M/5F)	19 to 26	Physically active Sport Science and Physiotherapy students	10 weeks 2 days/week 20 sessions 2980 jumps		Achilles tendon CSA	Achilles tendon stiffness	CMJ height DJ 40 cm height		MVC
Monti et al. [56]	*N* = 8 (M)	25.3 ± 4.6	Healthy, fully independent and recreationally active volunteers	6 weeks 3 days/week 18 sessions 20–25 min/session 2160–2700 jumps	VL CSA VL Pennation angle VL Fascicle length					MVC
Ogiso and Miki [64]	*N *= 23 (M) EMS = 8 Non-EMS = 9 CG = 6	EMS 21.0 ± 0.8 Non-EMS 19.2 ± 0.8 CG 21.7 ± 0.5	Healthy men with no orthopaedic or neuromuscular disorders and who train regularly	3 weeks 3 days/week 9 sessions			Achilles tendon stiffness	CMJ height SJ height DJ height		MVC torque 90 º
Paleckis et al. [26]	*N* = 9 (M)	21.6 ± 3.2	Healthy physically active untrained young men	9 days daily 9 sessions				CMJ height		MVC of quadriceps femoris
Potach et al. [69]	*N* = 16 (4 M/12F) PT = 8 (2 M/6F) CG = 8 (2 M/6F)	Not defined	College students	4 weeks 2 days/week 8 sessions				CMJ height		
Stien et al. [57]	*N* = 52 (F) OS = 18 17 OL = 18 17 CG = 16	21.2 ± 1.7	Resistance-trained women students	8 weeks 2–3 days/week 16–24 sessions 1380 jumps	VL Muscle thickness					
Taube et al. [70]	*N* = 33 (19 M/14F) SSC1 = 11 (7 M/4F) SSC2 = 11 (7 M/4F) CG = 11 (5 M/6F)	SSC1 24 ± 3 SSC2 25 ± 4 CG 24 ± 3	Healthy subjects participated in sporting activities containing jumps	4 weeks 3 days/week 12 sessions 45–60 min/session				DJ height		
Ullrich et al. [58]	*N* = 22 (12 M/10F) TP = 11 (6 M/5F) DUP = 11 (6 M/5F)	24.3 ± 2.6	Amateur athletes (soccer, handball, basketball, tennis and field hockey players)	6 weeks 3 days/week 18 sessions 40 min/session 540 jumps	VL Muscle thickness RF Muscle thickness VL Pennation angle RF Pennation angle VL Fascicle length RF Fascicle length			CMJ height		Bilateral leg extension MVC 90º
Van der Zwaard et al. [62]	*N* = 21 (F) APL = 7 no APL = 19	26 ± 4	Elite female rowers of the national team	16 weeks 1 day/week 16 sessions	VL Fascicle length VL Pennation angle					
Wu et al. [39]	*N* = 21 (M) PT = 11 CG = 10	PT 22.1 ± 1.6 CG 22.3 ± 1.6	Students at university student centre	8 weeks 2 days/week 16 sessions			Achilles tendon stiffness	CMJ height		
Zubac and Simunic [71]	*N* = 20 (10 M/10F) PT = 10 CG = 10	22.4 ± 4.7	Healthy active individuals for more than 5 h per week	8 weeks 3 days/week 24 sessions 45 min/session				CMJ height		

Abbreviations: APL, Additional plyometric loading; BFR, Jump Training with Blood Flow Restriction; BMSJT, Body Mass Squat Jump Training; CG, Control Group; CMJ, Countermovement Jump; CSA, Cross Sectional Area; DJ, Drop Jump; DUP, Daily Undulating Periodisation; ECR, Eccentric Calf Raise Exercise Group; EG, Experimental Group; EMS, Electromyostimulation; ET, Endurance Training; F, Female; FHS, Forward Hack Squat Training; FSPT, Free Weight Strength and Power Training; IP, Incline Plyometrics Group; ISO/ECC, Isokinetic Resistance Training and Eccentric Overload; IT, Isometric Training; M, Male; MSPT, Motorised Strength and Power Training; MVC, Maximal Voluntary Contraction; NCR, Normal Calf Raise Exercise Group; OL, Plyometric Training Utilising Overspeed; OM, Older Males; OS, Plyometric Training Utilising Overload; OWL, Olympic-style Weightlifting; P, Power Training to Increase Vertical Jump Height; PG, Power Group; PP, Plane Plyometrics; PS, Combination of Power and Stretch Training; PT, Plyometric Training Group; RF, Rectus Femoris; RG, Rapid Strength Group; RM, Maximum Repetition; S, Stretching to Increase Flexibility; SJ, Squat Jump; SJT, Sprint/jump Training Only; SQ, Squat Lift Training; SSC1, Stretch–shortening cycle doing drop jumps from 30, 50, and 75 cm drop heights; SSC2, Stretch–shortening cycle doing drop jumps exclusively from 30 cm; TG, Traditional Group; TG KE, Knees Extended Training Group; TG KF, Knees Flexed Training Group; TP, Traditional Periodisation; TRAD, Traditional Resistance Training; TS, Triceps Surae; VL, Vastus lateralis; VM, Vastus Medialis; WJST, Weighted Jump Squat; WT, Weight Training; YM, Young Males

### Statistical Analyses

Pre- and post-intervention mean ± standard deviation (SD) for outcomes from the PT groups were collected. Four meta-analyses were performed using Review Manager software (RevMan. Version 5.3. Copenhagen: Nordic Cochrane Centre, Cochrane Collaboration, 2014) for statistical analysis of the extracted data. Four meta-analyses were conducted: (1) muscle architecture; (2) tendon structure; (3) muscle and tendon stiffness; and (4) physical performance. The chi-square test and the Higgins *I*^2^ test were used to assess the heterogeneity among studies [44]. I^2^ ranges between 0 and 100%, where 0% indicates no observed heterogeneity, and larger values show increasing heterogeneity. The relationship between heterogeneity levels and *I*^2^ values is as follows: low level < 25%, moderate level 25–75% and high level > 75% of heterogeneity [45, 46]. A random-effects model using the Mantel–Haenszel method was used to pool the results of the different studies. Pooled odds ratios (OR) with 95% confidence intervals (CI) were calculated for the studies included in each meta-analysis. The standardised mean difference (SMD) and a 95% CI were also used for the analysis of continuous data [47]. The score of SMD was interpreted as follows: trivial: < 0.2, small effect: 0.2–0.5, moderate effect: 0.51–0.8, large effect: > 0.8 [48]. The *Z*-statistic (*Z*) was employed to analyse the overall effect. The significance criterion for all statistical tests was set at *p* < 0.05.

## Results

### Study Selection

**Fig. 1:**
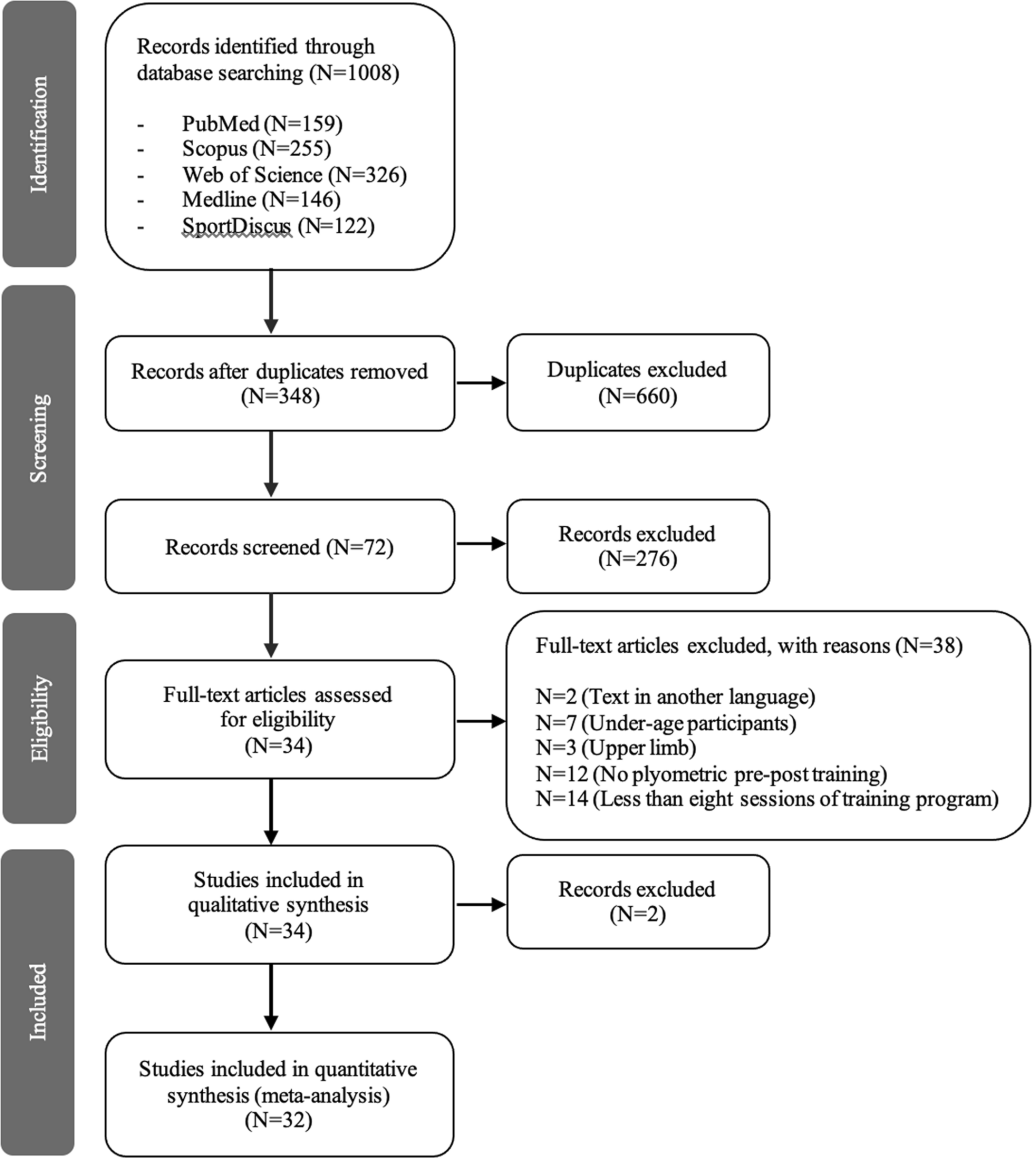
PRISMA flow diagram

The search of the different electronic databases identified 1008 articles. A total of 660 duplicates were removed and the remaining 348 titles and abstracts were reviewed. After reading the titles and abstracts, 72 articles remained. The full text of these 72 articles was retrieved and assessed for eligibility. Of the 72, 38 were excluded as not meeting the inclusion criteria and 34 studies were analysed. Data were requested for statistical analysis for some of the studies and were not available for 2 studies. Finally, 32 articles were included in the meta-analysis (Fig. 1).

This review focused on the evaluation of PT and its effects on lower body muscle architecture, tendon structure, muscle and tendon stiffness and different physical performance variables such as jump height (Counter Movement Jump (CMJ), Squat Jump (SJ) and Drop Jump (DJ)), velocity and strength. The thirty-two articles included in this review with meta-analysis used a variety of PT programmes. The frequency, intensity, duration, mode and sequence of the exercises and the design of the intervention differed among the studies (Table 1). Fifteen of the included studies analysed the effects of PT on muscle architecture [18, 49–62], eight articles investigated the effects on tendon structure [18, 25, 27, 28, 38, 54, 59, 61], thirteen studies evaluated the effects on muscle–tendon stiffness [18, 25, 27, 28, 36–39, 54, 59, 61, 63, 64], and twenty-nine showed the effects on physical performance [18, 25–28, 36–39, 49–54, 56, 58–61, 63–71].

The first meta-analysis on muscle architecture was structured into four subcategories: muscle thickness, fascicle length, CSA and pennation angle. In turn, each subcategory included the studies according to the muscle evaluated. For muscle thickness meta-analysis eleven studies [49–55, 57, 58, 60, 61] were included. In fascicle length and CSA meta-analysis, nine [18, 49–52, 55, 56, 58, 62] and three papers [18, 56, 59] were evaluated, respectively. These nine studies [18, 49–52, 55, 56, 58, 62] were also included for pennation angle meta-analysis. The meta-analysis on tendon structure included eight studies [18, 25, 27, 28, 38, 54, 59, 61] which analysed the Achilles tendon CSA. For the meta-analysis of stiffness, the selected studies were divided into two subcategories: muscle stiffness and tendon stiffness. For muscle stiffness three papers [36, 54, 59] were included and thirteen studies [18, 25, 27, 28, 36–39, 54, 59, 61, 63, 64] were analysed in the tendon stiffness meta-analysis. The last meta-analysis studied the effects of PT on three lower body physical performance variables: jumping, sprinting and lower body strength. For jump performance twenty-four papers [18, 25–28, 36–39, 49, 50, 52–54, 58, 60, 61, 64–67, 69–71] were analysed and divided into three categories according to the type of jump: CMJ (with twenty studies [18, 25–28, 38, 39, 49, 50, 52–54, 58, 60, 61, 64, 66, 67, 69, 71]), SJ (eleven studies [18, 25, 28, 36–38, 50, 52, 61, 64, 66]) and DJ (ten studies [27, 28, 52–54, 61, 64, 65, 67, 70]). In sprint performance meta-analysis five studies [25, 49, 50, 52, 53] were included, and twenty-one studies [26–28, 36–38, 49–54, 56, 58–60, 63–66, 68] were included for lower body strength performance meta-analysis.

### Methodological Quality Assessment

**Fig. 2:**
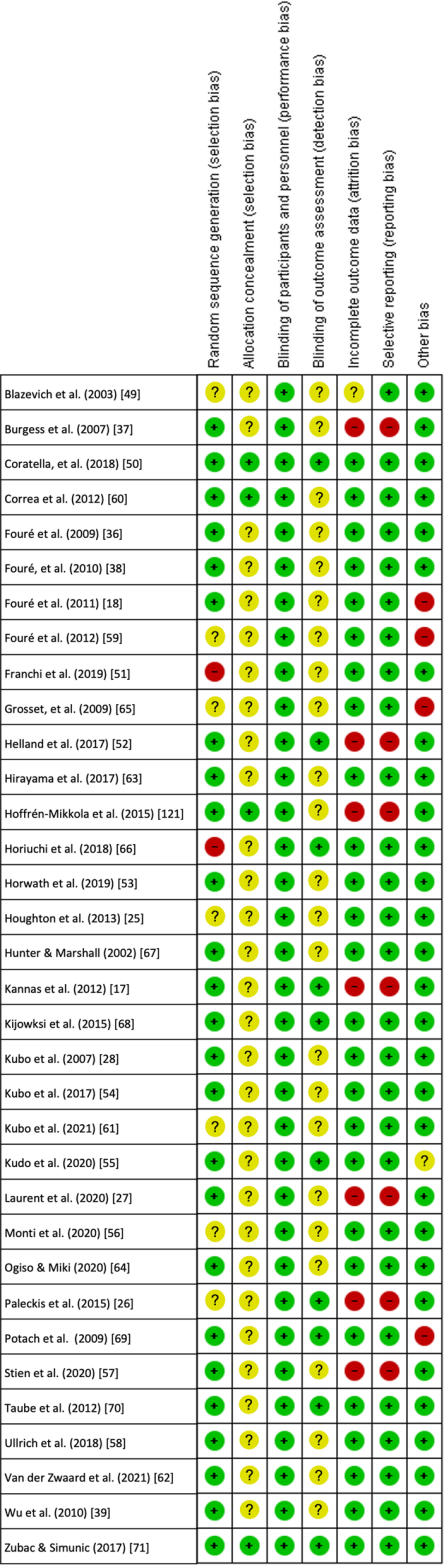
Risk of bias overall judgment. Note: When a study scores a " + " in all subdomains, the overall judgement is "low risk of bias". When a study scores "?" on one or more subdomains, the overall judgement is "some concerns". When a study scores a "-" in one or more subdomains, the overall assessment is "high risk of bias", giving rise to substantial doubts about the quality of the research

Methodological quality scores on the PEDro scale ranged from 3 to 8 (5.29 ± 1.14) out of a maximum of 10 points. Therefore, the set of studies was considered to be of moderate methodological quality. The most frequent biases were blinding of subjects (criterion 5), followed by blinding of therapists (criterion 6) and concealed allocation (criterion 3). Details of the PEDro scale for each study can be found in Appendix B. The risk of bias assessment showed a “high risk of bias” in twelve of the thirty-four included studies, twenty studies scored “some concern” and two papers were considered “low risk of bias” (Table 2, Fig. 2). The details of the risk of bias assessment of the included trials are shown in Appendix C. Overall, the risk of bias in the trials included in this meta-analysis was "some concerns".

**Table 2:** Risk of bias overall judgment

Study	Risk of bias
Blazevich et al. [49]	Some concerns
Burgess et al. [37]	High risk
Coratella, et al. [50]	Low risk
Correa et al. [60]	Some concerns
Fouré et al. [36]	Some concerns
Fouré, et al. [38]	Some concerns
Fouré et al. [18]	High risk
Fouré et al. [59]	High risk
Franchi et al. [51]	High risk
Grosset, et al. [65]	High risk
Helland et al. [52]	High risk
Hirayama et al. [63]	Some concerns
Hoffrén-Mikkola et al. [121]	High risk
Horiuchi et al. [66]	High risk
Horwath et al. [53]	Some concerns
Houghton et al. [25]	Some concerns
Hunter and Marshall [67]	Some concerns
Kannas et al. [17]	High risk
Kijowksi et al. [68]	Some concerns
Kubo et al. [28]	Some concerns
Kubo et al. [54]	Some concerns
Kubo et al. [61]	Some concerns
Kudo et al. [55]	Some concerns
Laurent et al. [27]	High risk
Monti et al. [56]	Some concerns
Ogiso and Miki [64]	Some concerns
Paleckis et al. [26]	High risk
Potach et al. [69]	High risk
Stien et al. [57]	Some concerns
Taube et al. [70]	Some concerns
Ullrich et al. [58]	Some concerns
Van der Zwaard et al. [62]	Some concerns
Wu et al. [39]	Some concerns
Zubac and Simunic [71]	Low risk

### Meta-analysis Results

#### Effects of Plyometric Training on Muscle Architecture

**Fig. 3:**
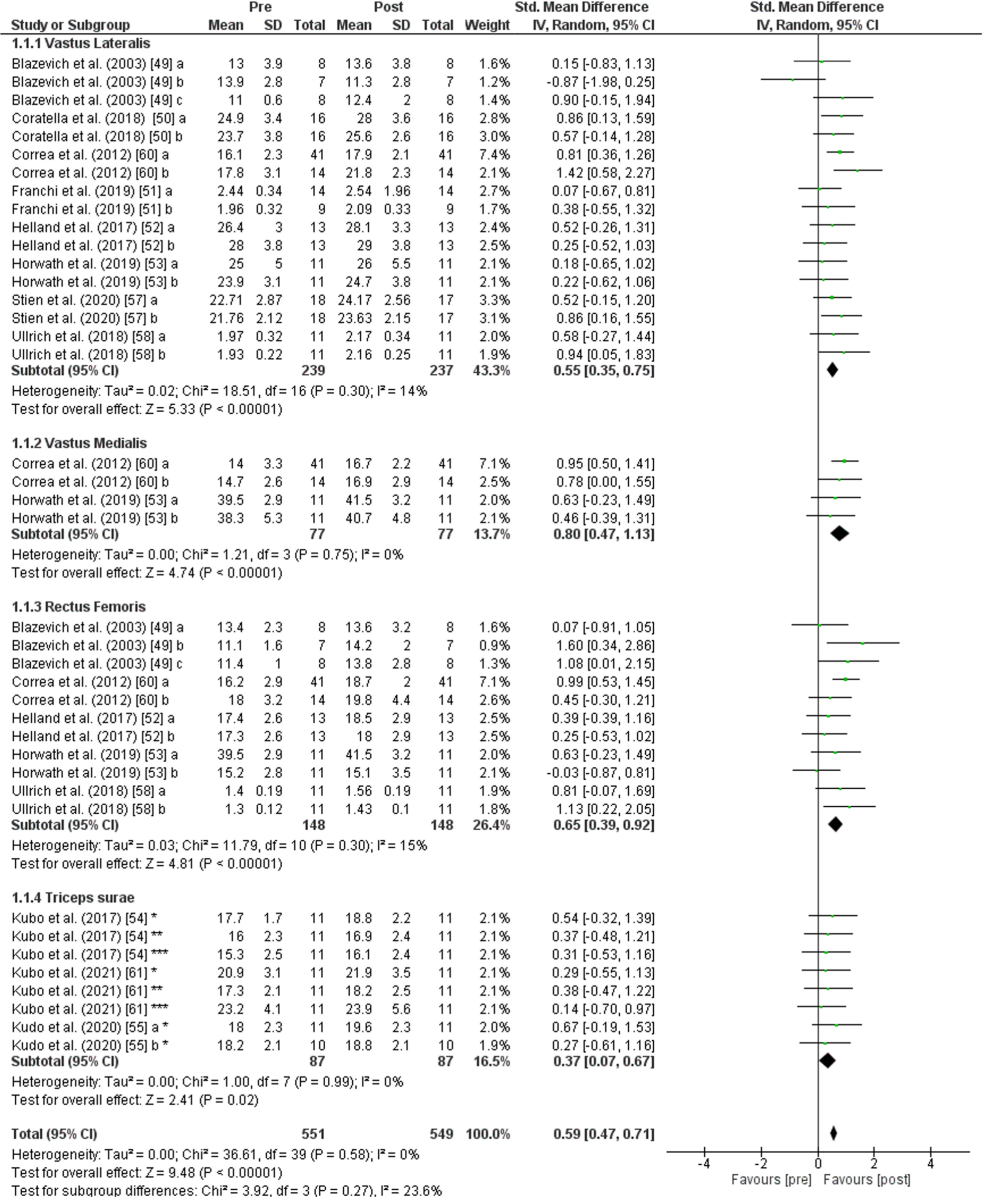
Effects of plyometric training on *muscle thickness.* Note: a = plyometric group 1; b = plyometric group 2; c = plyometric group 3; * = medial gastrocnemius; ** = lateral gastrocnemius; *** = soleus

The PT showed an increase (*p* < 0.001) of *muscle thickness* with moderate effect (SMD: 0.59; [95% CI 0.47, 0.71]; *n* = 549; *Z* = 9.48) and low heterogeneity (*I*^2^ = 0%). The subgroup analysis showed low heterogeneity (*I*^2^ = 23.6%) and non-significant differences (*p* = 0.270). Greater values of *vastus lateralis muscle thickness* were observed after PT (*p* < 0.001) with moderate effect (SMD: 0.55; [95% CI 0.35, 0.75], *n* = 237, *Z* = 5.33) and low heterogeneity (*I*^2^ = 14%). An increase of muscle thickness was also found after PT for the *vastus medialis muscle* (*p* < 0.001) with moderate effect (SMD: 0.80; [95% CI 0.47, 1.13], *n* = 77, *Z* = 4.74) and low heterogeneity (*I*^2^ = 0%), for *rectus femoris muscle* (*p* < 0.001) with moderate effect (SMD: 0.65; [95% CI 0.39, 0.92], *n* = 148, *Z* = 4.81) and low heterogeneity (*I*^2^ = 15%) and, for the *triceps surae muscle* (*p* = 0.020) with small effect (SMD: 0.37; [95% CI 0.07, 0.67], *n* = 87, *Z* = 2.41) and low heterogeneity (*I*^2^ = 0%) (Fig. 3).

**Fig. 4:**
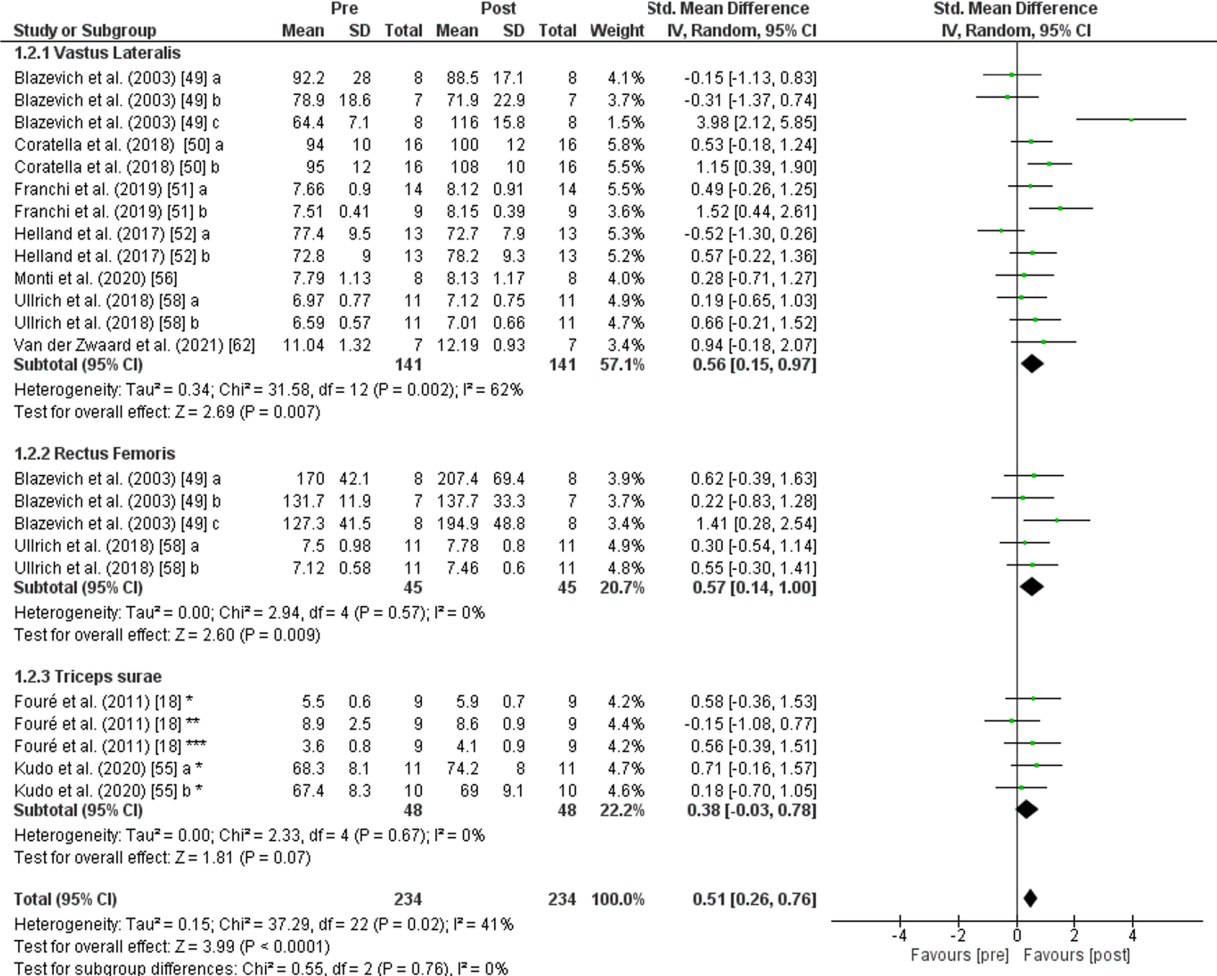
Effects of plyometric training on *fascicle length.* Note: a = plyometric group 1; b = plyometric group 2; c = plyometric group 3; * = medial gastrocnemius; ** = lateral gastrocnemius; *** = soleus

The PT showed an increase (*p* < 0.001) of *fascicle length* with moderate effect (SMD: 0.51; [95% CI 0.26, 0.76]; *n* = 234; *Z* = 3.99) and moderate heterogeneity (*I*^2^ = 41%). The subgroup analysis showed low heterogeneity (*I*^2^ = 0%) and non-significant differences (*p* = 0.760). Greater values of *vastus lateralis fascicle length* were observed after PT (*p* = 0.007) with moderate effect (SMD: 0.56; [95% CI 0.15, 0.97], *n* = 141, *Z* = 2.69) and moderate heterogeneity (*I*^2^ = 62%). An increase of *fascicle length* was also found after PT for the *rectus femoris muscle* (*p* = 0.009) with moderate effect (SMD: 0.57; [95% CI 0.14, 1.00], *n* = 45, *Z* = 2.60) and low heterogeneity (*I*^2^ = 0%). No change was recorded after PT in *fascicle length* of the *triceps surae muscle* (*p* = 0.070) with small effect (SMD: 0.38; [95% CI − 0.03, 0.78], *n* = 48, *Z* = 1.81) and low heterogeneity (*I*^2^ = 0%) (Fig. 4).

**Fig. 5:**
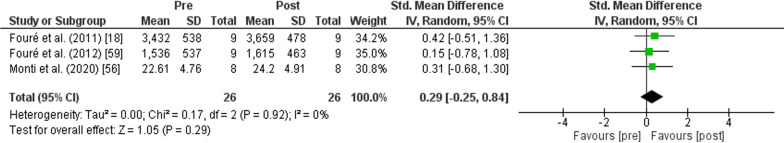
Effects of plyometric training on *cross-sectional area*

The PT showed no change in *CSA muscle* (*p* = 0.290) with small effect (SMD: 0.29; [95% CI − 0.25, 0.84]; *n* = 26; *Z* = 1.05) and low heterogeneity (*I*^2^ = 0%) (Fig. 5).

**Fig. 6:**
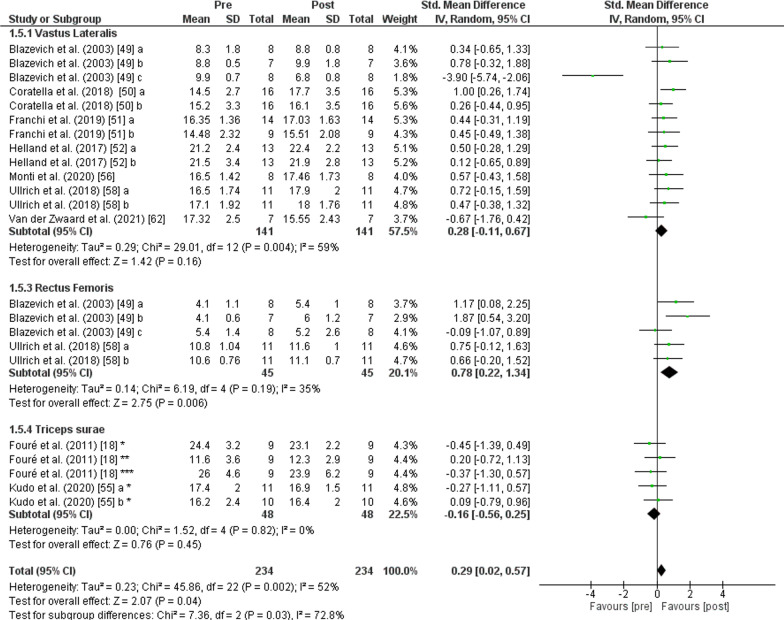
Effects of plyometric training on *pennation angle.* Note: a = plyometric group 1; b = plyometric group 2; c = plyometric group 3; * = medial gastrocnemius; ** = lateral gastrocnemius; *** = soleus

The PT showed an increase (*p* = 0.030) of *pennation angle* with small effect (SMD: 0.29; [95% CI 0.02, 0.57]; *n* = 234; *Z* = 2.07) and moderate heterogeneity (*I*^2^ = 52%). The subgroup analysis showed moderate heterogeneity (*I*^2^ = 72.8%) and significant differences (*p* = 0.030). An increase of *pennation angle* was also found after PT for the *rectus femoris muscle* (*p* = 0.006) with moderate effect (SMD: 0.78; [95% CI 0.22, 1.34], *n* = 45, *Z* = 2.75) and moderate heterogeneity (*I*^2^ = 35%). No change was recorded after PT in the *pennation angle of the vastus lateralis* (*p* = 0.160) with small effect (SMD: 0.28; [95% CI − 0.11, 0.67], *n* = 141, *Z* = 1.42) and moderate heterogeneity (*I*^2^ = 59%) and for the *triceps surae muscle* (*p* = 0.450) with trivial effect (SMD: − 0.16; [95% CI − 0.56, 0.25], *n* = 48, *Z* = 0.76)) and low heterogeneity (*I*^2^ = 0%) (Fig. 6).

#### Effects of Plyometric Training on Tendon Structure

**Fig. 7:**
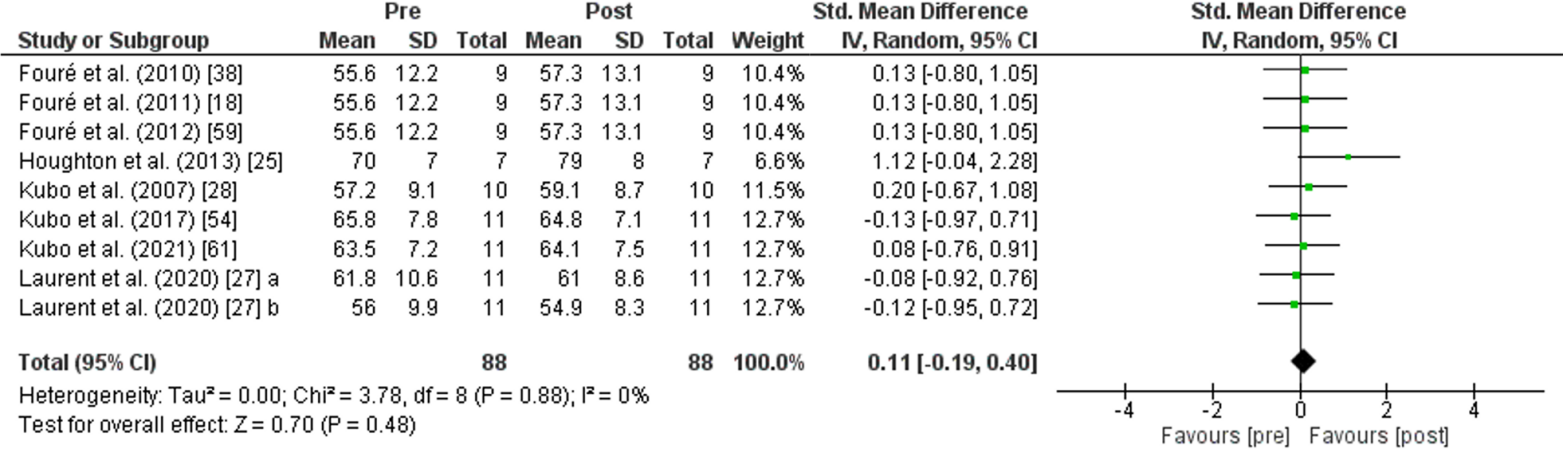
Effects of plyometric training on *tendon structure*. Note: a = plyometric group 1; b = plyometric group 2

The PT showed no change in *CSA of Achilles tendon* (*p* = 0.480) after PT with trivial effect (SMD: 0.11; [95% CI − 0.19, 0.40]; *n* = 88; *Z* = 0.70) and low heterogeneity (*I*^2^ = 0%) (Fig. 7).

#### Effects of Plyometric Training on Muscle and Tendon Stiffness

**Fig. 8:**
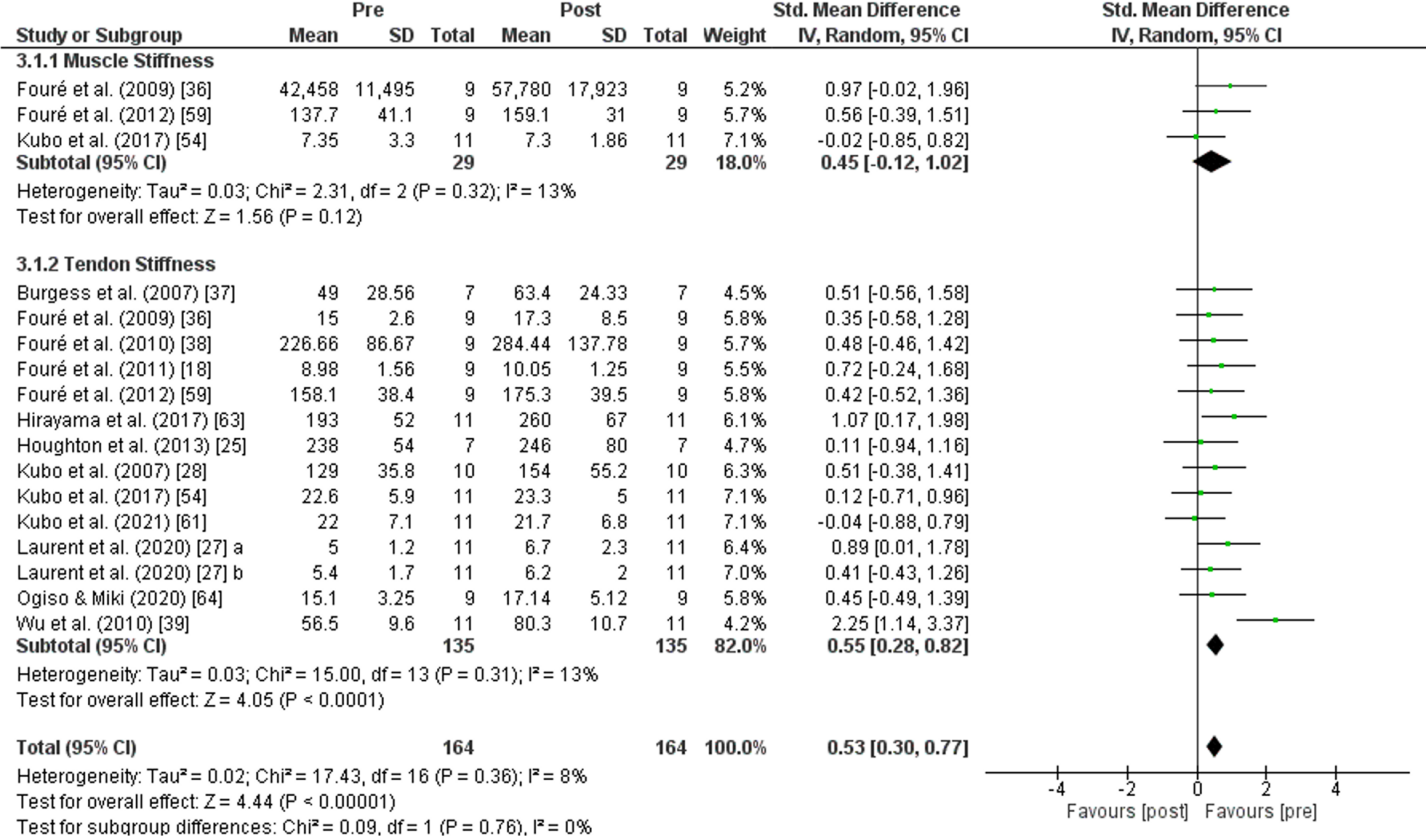
Effects of plyometric training on *muscle and tendon stiffness.* Note: a = plyometric group 1; b = plyometric group 2

The PT showed an increase (*p* < 0.001) of *stiffness* with moderate effect (SMD: 0.53; [95% CI 0.33, 0.77]; *n* = 164; *Z* = 4.44) and low heterogeneity (*I*^2^ = 8%). The subgroup analysis showed low heterogeneity (*I*^2^ = 0%) and non-significant differences (*p* = 0.760). No change was recorded after PT in *muscle stiffness* (*p* = 0.120) with small effect (SMD: 0.45; [95% CI − 0.12, 1.02], *n* = 29, *Z* = 1.56) and low heterogeneity (*I*^2^ = 13%). An increase of *tendon stiffness* was also found after PT (*p* < 0.001) with moderate effect (SMD: 0.55; [95% CI 0.28, 0.82], *n* = 135, *Z* = 4.05) and low heterogeneity (*I*^2^ = 13%) (Fig. 8).

#### Effects of Plyometric Training on Lower Body Physical Performance

**Fig. 9:**
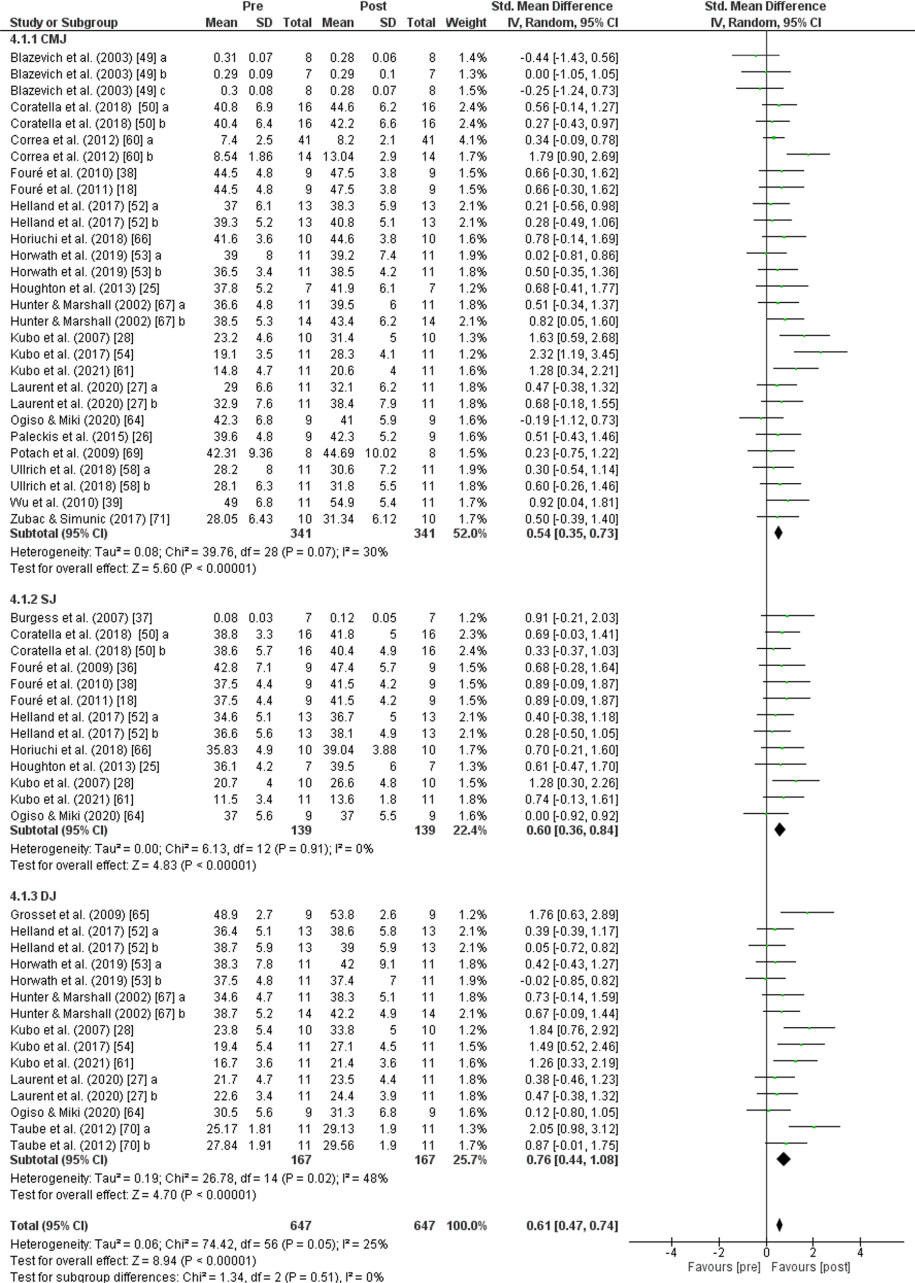
Effects of plyometric training on *jump performance.* Note: a = plyometric group 1; b = plyometric group 2; c = plyometric group 3

**Fig. 10:**
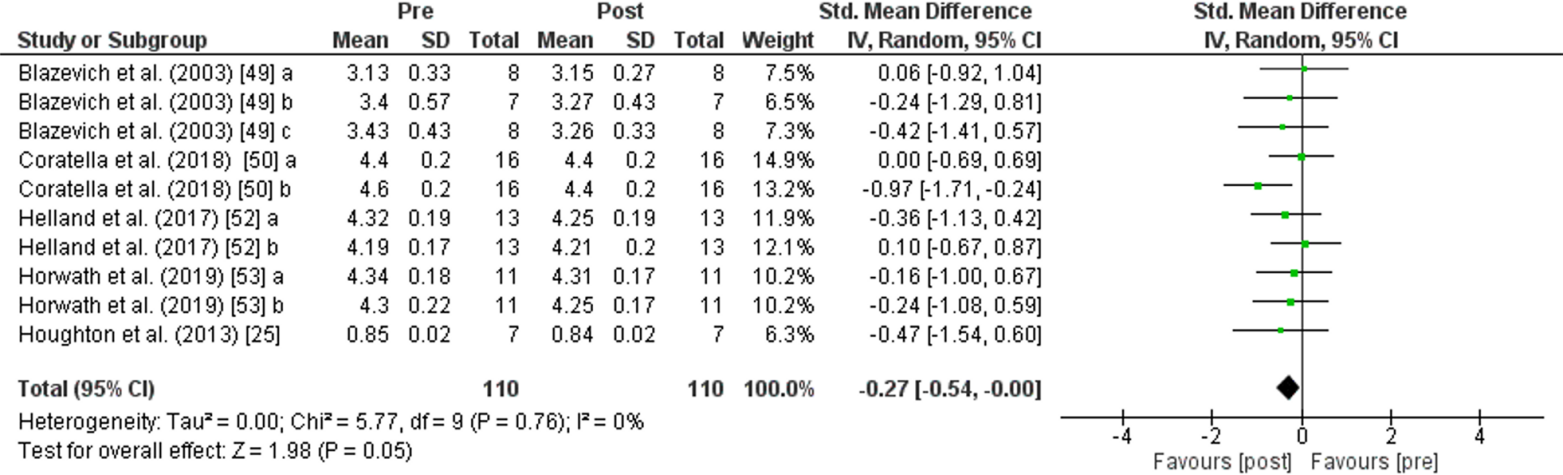
Effects of plyometric training on *sprint performance.* Note: a = plyometric group 1; b = plyometric group 2; c = plyometric group 3

**Fig. 11:**
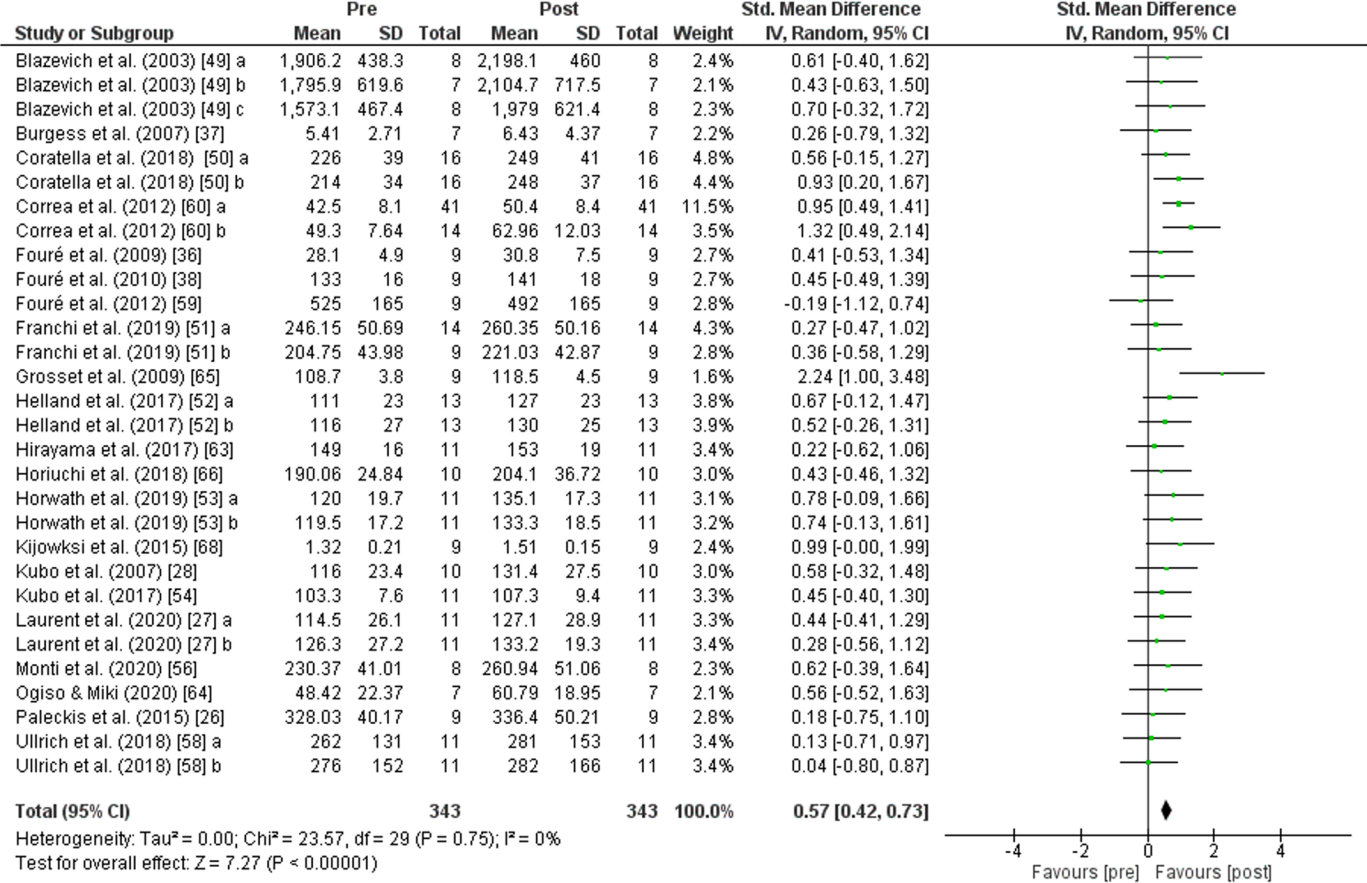
Effects of plyometric training on lower body *strength performance.* Note: a = plyometric group 1; b = plyometric group 2; c = plyometric group 3

The PT showed an increase (*p* < 0.001) of *jump performance* with moderate effect (SMD: 0.61; [95% CI 0.47, 0.74]; *n* = 647; *Z* = 8.94) and moderate heterogeneity (*I*^2^ = 25%). The subgroup analysis showed low heterogeneity (*I*^2^ = 0%) and non-significant differences (*p* = 0.510). An increase in *jump height* was also found after PT for *CMJ* (*p* < 0.001) with moderate effect (SMD: 0.54; [95% CI 0.35, 0.73], *n* = 341, *Z* = 5.60) and moderate heterogeneity (*I*^2^ = 30%), for *SJ* (*p* < 0.001) with moderate effect (SMD: 0.60; [95% CI 0.36, 0.84], *n* = 139, *Z* = 4.83) and low heterogeneity (*I*^2^ = 0%), and for *DJ* (*p* < 0.001) with moderate effect (SMD: 0.76; [95% CI 0.44, 1.08], *n* = 167, *Z* = 4.70) and moderate heterogeneity (*I*^2^ = 48%) (Fig. 9). No change in *sprint performance* was observed after PT (*p* = 0.050) with small effect (SMD: − 0.27; [95% CI − 0.54, − 0.00]; *n* = 110; *Z* = 1.98) and low heterogeneity (*I*^2^ = 0%) (Fig. 10). The PT showed an increase (*p* < 0.001) of lower body *strength performance* with moderate effect (SMD: 0.57; [95% CI 0.42, 0.73]; *n* = 343; *Z* = 7.27) and low heterogeneity (*I*^2^ = 0%) (Fig. 11).

## Discussion

This systematic review and meta-analysis aimed to assess the effects of PT on lower body muscle architecture, tendon structure, muscle–tendon stiffness and physical performance. From records we retrieved, 32 studies were eligible for inclusion in the meta-analysis. The main findings of our study were that PT increased the thickness of different muscles in the lower limbs as well as an increase in the pennation angle of rectus femoris, and fascicle length of the vastus lateralis and rectus femoris. Furthermore, tendon stiffness increased and improvements in jump and lower body strength performance were also recorded after PT programmes.

### Effects of Plyometric Training on Muscle Architecture

For *muscle thickness* we analysed the effects of PT on four muscles of the lower limb: vastus lateralis, vastus medialis, rectus femoris and triceps surae, and we found an increase in the thickness of these four muscles. Previous studies indicated that eccentric exercise provokes the increase of fascicle length [72], so the increase of the fascicle length of the vastus lateralis and rectus femoris could indicate that the eccentric load of the plyometric exercises would be supported by the quadriceps muscle. For the *CSA* of the different muscles analysed (vastus lateralis and triceps surae), we found no significant differences after a PT programme. For this analysis of *CSA*, it is worth noting the paucity of studies was found, as well as the number of subjects analysed. Early responses in muscle *CSA* may be influenced by oedema provoked by the eccentric component of exercise in early PT sessions [56].

Notably, the exercise-related adaptations of *pennation angle* and *fascicle length* could result in increases of muscle thickness [49]. These architectural changes in muscle play an important role in increasing force production [73]. Increases in *fascicle length* have been observed following periods of isometric [74], concentric [75, 76] and eccentric exercise [76, 77], with the increases being greater with heavier loads during eccentric exercise [76, 78]. These increases in *fascicle length* can affect a muscle's strength-to-length ratio and strength-to-velocity ratio [49, 75, 79] and may also prevent muscle injury during explosive movements [79]. In addition, an increase in *pennation angle* may reflect the addition of sarcomeres in parallel [80] and an increase in *fascicle length* is indicative of a potential addition of sarcomeres in series [76, 81]. Therefore, it is hypothesised that increases in *fascicle length* may be induced by the imposition of stresses on the fibres/fascicles [82].

On the other hand, these different results found in the changes of muscle architecture could be due to the different training protocols carried out [50, 57] or even to the different populations involved [50]. The effects of PT may differ according to the different characteristics of the subjects such as: sex and age [83], training level [84, 85], and physical activity performed or even familiarity with plyometric training [86, 87]. It is important to bear in mind that this combination of variables may lead to contradictory results. It is to be expected that less fit individuals are more likely to improve their muscle architecture and make greater gains during the first few weeks of training than people with a higher level of fitness [88]. An increase in efferent neural drive could be the explanation for the greater changes in less experienced individuals according to the study by Aagaard et al. [89]. Regarding the training protocol, factors such as programme duration, intensity and training volume could determine the effectiveness of the PT for the adaptations to be observed [87]. Numerous authors have included different combinations of these factors in their PT protocols [5, 90, 91], but the ideal combination to achieve the best gains remains unclear. Some research which applied PT programmes with strength exercises (i.e. squats, dead lifts) found the greatest increases of *muscle thickness* [49, 92].

### Effects of Plyometric Training on Tendon Structure

No statistically significant changes were found for tendon structure after a PT programme. However, a small increase in *Achilles tendon CSA* is observed after PT. This increase could be due to reactivated tendinopathy (temporary changes) or reflect permanent hypertrophy of the Achilles tendon [93]. Some cross-sectional studies suggest that a history of repetitive lower limb loading is associated with increased *Achilles tendon CSA*, especially in the distal region [94]. Therefore, adequate mechanical loading can cause positive changes in tendon structures and lead to improved performance, but also excessive loading can induce tendon degeneration [95]. This could be the answer to the lack of significant results in the studies by Fouré et al. [18, 38, 59] and Kubo et al. [28, 54] as they were longer and more intense interventions (12–14 weeks and 34–48 sessions). In contrast, the study by Houghton et al. [25] whose training programme duration and intensity were shorter (8 weeks and 16–24 sessions) showed the greatest increase in *Achilles tendon CSA* in their results. Another reason could be the one stated by Fouré et al. [38], who considered that the change in *Achilles tendon CSA* could have been undetectable in their study, because the CSA measurement was taken at the medial level of the Achilles tendon and not in the distal region as Magnusson and Kjaer [94] claimed. Finally, it should be noted that the lack of change in *CSA*, combined with increased maximal voluntary contraction and subsequent tendon stress, may predispose the tendon to injury (i.e. rupture and tendinopathy) [38]. Therefore, in order to increase the *CSA* of the tendon and avoid tendon degeneration that may lead to injury, it would be interesting for future research to find the boundary between an adequate mechanical load and an excessive mechanical load in a PT programme.

### Effects of Plyometric Training on Muscle and Tendon Stiffness

Our results show a significant increase in stiffness after a PT programme. The type of training could change the elastic behaviour of the soft tissues that make up the joint (muscle and tendon) [7]. Some authors suggest that a stiff muscle–tendon complex is necessary for the optimal performance of SSC activities [96–99], since it allows a faster and efficient transmission of muscle force to the skeleton, increasing rates of force development**.** In the separate analysis of the adaptations of *muscle stiffness* and *tendon stiffness* to PT, we found contradictory results, as *muscle stiffness* did not show any change after PT programmes, but *tendon stiffness* did. The reason could be that elastic energy accumulates more in the tendons than in muscle fibres [100]. Another reason could be the difference in the number of total subjects (*n* = 29 for *muscle stiffness* and *n* = 135 for *tendon stiffness*).

The results show significant increases in *tendon stiffness* following a PT programme. Many studies have shown that PT leads to an improvement in the mechanical properties of the tendon, understood as an increase in its stiffness [27, 37, 38]. When a muscle–tendon unit is repeatedly exposed to increased mechanical loading, muscle strength gains are observed to be accompanied by an increase in *tendon stiffness* [101–103]. This can be seen in the eight studies [27, 28, 36–38, 54, 59, 63] that assessed both *tendon stiffness* and lower body strength performance after PT. All of them found an increase in both strength and *tendon stiffness.*

Finally, *muscle stiffness* results showed no significant differences as an effect of PT. Greater *muscle stiffness* has the advantage of allowing greater storage, release and efficient reuse of elastic energy in SSC activities [104, 105]. The results obtained in the study by Ikezoe et al. [106] showed that *muscle stiffness* was significantly associated with muscle thickness, and in turn, a relationship between muscle thickness and muscle strength is observed, which is consistent with previous studies showing that muscle strength increased linearly as muscle size increases. Therefore, to increase *muscle stiffness*, it will be critical to increase the force-producing capacity of the muscle [105]. Unfortunately, because the small number of studies found that looked at *muscle stiffness* in the lower extremity following PT, we cannot discuss this point with complete certainty.

### Effects of Plyometric Training on Lower Body Physical Performance

Our meta-analysis showed significant changes in *jump performance* (CMJ, SJ and DJ) after a PT programme. This gain in jumping can be attributed to factors such as improved recruitment of motor units, increased neural drive to agonist muscles, improved intermuscular coordination, better utilisation of the SSC [7] and possibly selective muscle hypertrophy [19]. The highest SMD was found in the DJ, which could be due to biomechanical and physiological differences among the types of jumps [107]. Thus, a substantial difference exists in the mechanical output and jump performance between slow SSC jumps (i.e. CMJ), fast SSC jumps (i.e. DJ) and concentric-only jumps (i.e. SJ) [108, 109]. In this meta-analysis, the studies that stand out most for their significant improvement [28, 54, 60, 65] have in common that they dealt with people with low physical activity, and therefore, their margin for improvement was greater than in the studies that worked with people who were already trained [88]. Furthermore, they carried out a PT programme lasting more than 10 weeks and 20 sessions, which would be in line with the recommendations of de Villarreal et al. [110], who demonstrated a positive relationship between the duration of the programme and the number of sessions with the effect of PT on *jump performance*, and recommend programmes lasting more than 10 weeks and with more than 20 sessions.

As for the results of our meta-analysis on *sprint performance*, we found a tendency to reduce the time in sprint after a PT programme, but no significant differences (*p* = 0.050) were found. Improvements in SSC efficiency and neuromechanical properties following a PT programme [7] contribute to the production of greater strength in the concentric phase of the movement after a fast eccentric muscle action [7, 111]. This is a fundamental requirement for improved *sprint performance* [112] and therefore a reason for the tendency to reduce the time in sprint after the PT programme found in our meta-analysis. Furthermore, it is hypothesised that greater improvements in sprint performance may be due to greater training specificity [113]. It is possible that a training programme that incorporates more horizontal acceleration (i.e. sprint-specific plyometric exercises, jumps with horizontal displacement) may significantly improve sprint times more than training programmes that include essentially vertical plyometric exercises [114]. Finally, it should be noted that the studies included in the meta-analysis on linear sprinting are few and their participants were considered athletes, most of them belonging to different sports clubs. This physical activity base, together with the scarcity of studies and the heterogeneity in the sprint test (20 m [49], 30 m [50, 53], and 5 m [25]), may have been decisive for the post-PT results. Therefore, more studies evaluating the effects of plyometrics on linear sprinting are needed to draw more solid conclusions.

The implementation of a PT programme showed significant improvements in different manifestations of lower body *strength*, such as concentric maximal strength and isometric maximal strength, as supported by previous studies [5, 87, 115]. In addition, there is evidence that PT improves muscular fitness (i.e. muscular strength, muscular power, local muscular endurance) [116–118]. Improvements in lower body *strength* after plyometric work are probably due to neural adaptations such as increased firing rate, synchronisation, excitability and efferent motor drive of motor units [7] and may also be related to muscle hypertrophy [19]. We highlight the results of the studies by Correa et al. [60] and Grosset et al. [65], who have the highest degree of improvement compared to the other studies. Both studies have in common that their subjects are people performing little or no physical activity and that they also carry out a training programme of more than 10 weeks and 20 sessions; therefore they would have a greater margin for improvement than the population that is already trained [88, 110]. However, there would be some studies where no significant differences are shown after the PT programme, which could be attributed to several reasons such as the nature of the training protocol, the type of plyometric and weight training exercises used and/or the training stimulus [87]. Some authors recommend combining training modalities (i.e. plyometrics and high-intensity resistance training) to optimise maximal strength gains, rather than using a single modality [119, 120]. Furthermore, training that combines plyometric exercises with additional weights has been shown to achieve greater gains in lower limb muscle *strength* [87].

### Study Limitations

Some potential limitations of this systematic review and meta-analysis should be acknowledged. The results are influenced by the heterogeneity of the studies, such as the characteristics of the participants or the different PT protocols (volume, intensity and duration of the programmes), which would limit direct comparisons among them. When a less fit person starts to exercise regularly, greater gains are usually achieved during the first few weeks compared to physically active people. This could be the reason for the larger changes for the same parameter in studies where individuals are less trained. On the other hand, volume (duration and number of training programme sessions) is a key aspect to take into account for the design of an optimal PT programme. In this meta-analysis, the training protocols were not exactly the same, although all included plyometric exercises, and therefore we could consider this as a limitation of the study. Another limitation for some of the meta-analyses was the small number of articles found (i.e. sprint or muscle CSA), which prevented firm conclusions on the effects of PT on these parameters. In addition, data from some studies that could have been included in the review were lost. Finally, the low scores of some of the studies on the Risk of bias assessment and the PEDro scale are noteworthy. These results are partly due to the criteria for the blinding of participants, therapists or evaluators, which makes the study score lower. However, we do not consider this as a risk of bias or poor study quality as it is a criterion that does not influence the final results due to our type of intervention.

## Conclusion

This systematic review with meta-analysis provides an overview of published studies on the effects of a PT programme on different parameters of lower body muscle architecture, tendon structure, muscle–tendon stiffness and physical performance, in different population types. In conclusion, a PT programme appears to be effective in increasing the muscle thickness of the vastus lateralis, vastus medialis, rectus femoris and triceps surae. It also provides significant changes in the fascicle length of vastus lateralis and rectus femoris muscles, and pennation angle of the rectus femoris muscle. In addition, plyometrics is considered an effective tool for increasing tendon stiffness and for producing improvements in jump performance (CMJ, SJ, DJ) and lower body strength performance.

### Practical Applications

PT can be recommended as a training modality to improve different parameters of lower body muscle architecture, stiffness or physical performance. The positive effects of PT are related to factors such as the characteristics of the subjects (age, sex, fitness level, etc.), but caution must be exercised because a combination of these variables can lead to contradictory results. The design of the training programme, the duration of the training and the volume of training are also considered key aspects to achieve favourable results after PT. Based on the studies that obtained the greatest improvements after training, our results show concordance with those reported by de Villarreal et al. [110] who recommend programmes of more than 10 weeks and with more than 20 sessions, and therefore seem to be the most indicated for the improvement of lower body physical performance. However, other studies suggest that a high load of this type of plyometric exercise may lead to deterioration of the tendon structure or even injury [38]. Therefore, these considerations should be taken into account by health and sport professionals to design an optimal PT programme.

## Figures and Tables

**Fig. 12:**
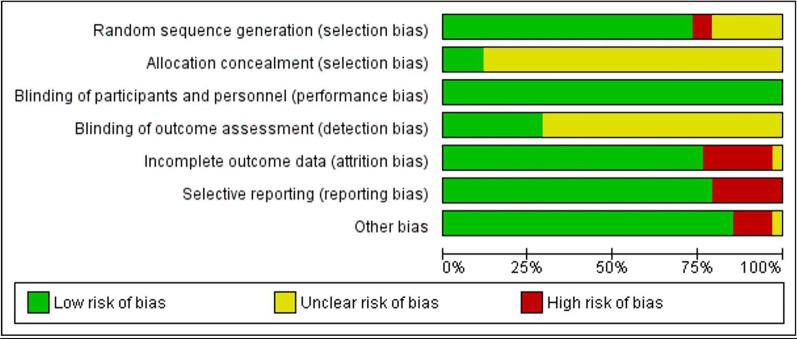
Risk of bias assessment of the included trials

## Data Availability

All data generated or analysed during this study are included in this published article [and its supplementary information files].

## Appendix A

### PUBMED (25/01/2022)

**Search:** (((((((("plyometrics"[Title/Abstract]) OR ("plyometric"[Title/Abstract])) OR ("pliometric"[Title/Abstract])) OR ("stretch–shortening cycle"[Title/Abstract])) OR ("drop jump"[Title/Abstract])) OR ("jump training"[Title/Abstract])) AND ((((("muscle architecture"[Title/Abstract]) OR ("physiological cross sectional area"[Title/Abstract])) OR ("fascicle length"[Title/Abstract])) OR ("pennation angle"[Title/Abstract])) OR ("muscle thickness"[Title/Abstract]))) NOT ("review"[Title/Abstract])) OR (((((((("plyometrics"[Title/Abstract]) OR ("plyometric"[Title/Abstract])) OR ("pliometric"[Title/Abstract])) OR ("stretch–shortening cycle"[Title/Abstract])) OR ("drop jump"[Title/Abstract])) OR ("jump training"[Title/Abstract])) AND (((("tendon"[Title/Abstract]) OR ("tendon structure"[Title/Abstract])) OR ("tendon cross sectional area"[Title/Abstract])) OR ("tendon thickness"[Title/Abstract]))) NOT ("review"[Title/Abstract])).

**Results: 159**

### SCOPUS (25/01/2022)

**Search:** ( ( ( TITLE-ABS-KEY ( "plyometrics") OR TITLE-ABS-KEY ( "plyometric") OR TITLE-ABS-KEY ( "pliometric") OR TITLE-ABS-KEY ( "stretch–shortening cycle") OR TITLE-ABS-KEY ( "drop jump") OR TITLE-ABS-KEY ( "jump training"))) AND ( ( TITLE-ABS-KEY ( "muscle architecture") OR TITLE-ABS-KEY ( "physiological cross sectional area") OR TITLE-ABS-KEY ( "fascicle length") OR TITLE-ABS-KEY ( "pennation angle") OR TITLE-ABS-KEY ( "muscle thickness"))) AND NOT ( TITLE-ABS-KEY ( "review"))) OR ( ( ( TITLE-ABS-KEY ( "plyometrics") OR TITLE-ABS-KEY ( "plyometric") OR TITLE-ABS-KEY ( "pliometric") OR TITLE-ABS-KEY ( "stretch–shortening cycle") OR TITLE-ABS-KEY ( "drop jump") OR TITLE-ABS-KEY ( "jump training"))) AND ( ( TITLE-ABS-KEY ( "tendon") OR TITLE-ABS-KEY ( "tendon structure") OR TITLE-ABS-KEY ( "tendon cross sectional area") OR TITLE-ABS-KEY ( "tendon thickness"))) AND NOT ( TITLE-ABS-KEY ( "review"))).

**Results: 255**

### WEB OF SCIENCE (25/01/2022)

**Search:** TS = ("plyometrics" OR "plyometric" OR "pliometric" OR "stretch–shortening cycle" OR "drop jump" OR "jump training") AND TS = ("muscle architecture" OR "physiological cross sectional area" OR "fascicle length" OR "pennation angle" OR "muscle thickness") NOT TS = ("review") OR TS = ("plyometrics" OR "plyometric" OR "pliometric" OR "stretch–shortening cycle" OR "drop jump" OR "jump training") AND TS = ("tendon" OR "tendon structure" OR "tendon cross sectional area" OR "tendon thickness") NOT TS = ("review").

**Results: 326**

### MEDLINE (25/01/2022)

**Search:** ( ( AB "plyometrics" OR TI "plyometrics" OR AB "plyometric" OR TI "plyometric" OR AB "pliometric" OR TI "pliometric" OR AB "stretch–shortening cycle" OR TI "stretch–shortening cycle" OR AB "drop jump" OR TI "drop jump" OR AB "jump training" OR TI "jump training") AND ( AB "muscle architecture" OR TI "muscle architecture" OR AB "physiological cross sectional area" OR TI "physiological cross sectional area" OR AB "fascicle length" OR TI "fascicle length" OR AB "pennation angle" OR TI "pennation angle" OR AB "muscle thickness" OR TI "muscle thickness") NOT ( AB "review" OR TI "review")) OR ( ( AB "plyometrics" OR TI "plyometrics" OR AB "plyometric" OR TI "plyometric" OR AB "pliometric" OR TI "pliometric" OR AB "stretch–shortening cycle" OR TI "stretch–shortening cycle" OR AB "drop jump" OR TI "drop jump" OR AB "jump training" OR TI "jump training") AND ( AB "tendon" OR TI "tendon" OR AB "tendon structure" OR TI "tendon structure" OR AB "tendon cross sectional area" OR TI "tendon cross sectional area" OR AB "tendon thickness" OR TI "tendon thickness") NOT ( AB "review" OR TI "review")).

**Results: 146**

### SPORTDISCUS (25/01/2022)

**Search:** ( ( AB "plyometrics" OR TI "plyometrics" OR AB "plyometric" OR TI "plyometric" OR AB "pliometric" OR TI "pliometric" OR AB "stretch–shortening cycle" OR TI "stretch–shortening cycle" OR AB "drop jump" OR TI "drop jump" OR AB "jump training" OR TI "jump training") AND ( AB "muscle architecture" OR TI "muscle architecture" OR AB "physiological cross sectional area" OR TI "physiological cross sectional area" OR AB "fascicle length" OR TI "fascicle length" OR AB "pennation angle" OR TI "pennation angle" OR AB "muscle thickness" OR TI "muscle thickness") NOT ( AB "review" OR TI "review")) OR ( ( AB "plyometrics" OR TI "plyometrics" OR AB "plyometric" OR TI "plyometric" OR AB "pliometric" OR TI "pliometric" OR AB "stretch–shortening cycle" OR TI "stretch–shortening cycle" OR AB "drop jump" OR TI "drop jump" OR AB "jump training" OR TI "jump training") AND ( AB "tendon" OR TI "tendon" OR AB "tendon structure" OR TI "tendon structure" OR AB "tendon cross sectional area" OR TI "tendon cross sectional area" OR AB "tendon thickness" OR TI "tendon thickness") NOT ( AB "review" OR TI "review")).

**Results: 122**

## Appendix B

See Table 3.

**Table 3:** Criterion 1 eligibility criteria were specified, Criterion 2 subjects were randomly allocated to groups, Criterion 3 allocation was concealed, Criterion 4 the groups were similar at baseline regarding the most important prognostic indicators, Criterion 5 there was blinding of all subjects, Criterion 6 there was blinding of all therapists who administered the therapy, Criterion 7 there was blinding of all assessors who measured at least one key outcome, Criterion 8 measures of at least one key outcome were obtained from more than 85% of the subjects initially allocated to groups, Criterion 9 all subjects for whom outcome measures were available received the treatment or control condition as allocated, Criterion 10 the results of between-group statistical comparisons are reported for at least one key outcome, Criterion 11 the study provides both point measures and measures of variability for at least one key outcome

Study	Criterion 1	Criterion 2	Criterion 3	Criterion 4	Criterion 5	Criterion 6	Criterion 7	Criterion 8	Criterion 9	Criterion 10	Criterion 11	TOTAL
Blazevich et al. [49]	1	0	0	1	0	1	0	0	1	1	1	5
Burgess et al. [37]	0	1	0	1	0	0	0	0	1	1	1	5
Coratella, et al. [50]	1	1	1	1	0	0	1	1	1	1	1	8
Correa et al. [60]	1	1	0	1	0	0	0	1	1	1	1	6
Fouré et al. [36]	0	1	0	1	0	0	0	1	1	1	1	6
Fouré, et al. [38]	0	1	0	1	0	0	0	0	1	1	1	5
Fouré et al. [18]	0	1	0	1	0	0	0	0	1	1	1	5
Fouré et al. [59]	0	0	0	1	0	0	0	0	1	1	1	4
Franchi et al. [51]	1	0	0	0	0	0	0	0	1	1	1	3
Grosset, et al. [65]	0	0	0	1	0	0	0	1	1	1	1	5
Helland et al. [52]	1	1	0	1	0	0	1	1	1	1	1	7
Hirayama et al. [63]	0	1	0	1	0	0	0	1	1	1	1	6
Hoffrén-Mikkola et al. [121]	1	1	1	1	0	0	0	1	1	1	1	7
Horiuchi et al. [66]	0	0	0	1	0	0	0	0	1	1	1	4
Horwath et al. [53]	1	1	0	1	0	0	0	0	1	1	1	5
Houghton et al. [25]	1	0	0	1	0	0	0	0	1	1	1	4
Hunter and Marshall [67]	0	1	0	1	0	0	0	1	1	1	1	6
Kannas et al. [17]	1	1	0	1	0	0	0	0	1	1	1	5
Kijowksi et al. [68]	1	1	0	1	0	0	0	0	1	1	1	5
Kubo et al. [28]	0	1	0	1	0	0	0	0	1	1	1	5
Kubo et al. [54]	0	1	0	1	0	0	0	0	1	1	1	5
Kubo et al. [61]	0	0	0	1	0	0	0	1	1	1	1	5
Kudo et al. [55]	1	1	0	1	0	0	0	1	1	1	1	6
Laurent et al. [27]	0	1	0	1	0	0	0	1	1	1	1	6
Monti et al. [56]	1	0	0	0	0	0	0	0	1	1	1	3
Ogiso and Miki [64]	0	1	0	1	0	0	0	1	1	1	1	6
Paleckis et al. [26]	0	0	0	0	0	0	0	0	1	1	1	3
Potach et al. [69]	1	1	0	1	0	0	1	1	1	1	1	7
Stien et al. [57]	1	1	0	1	0	0	0	1	1	1	1	6
Taube et al. [70]	1	1	0	1	0	0	0	0	1	1	1	5
Ullrich et al. [58]	1	1	0	1	0	0	0	1	1	1	1	6
Van der Zwaard et al. [62]	0	0	0	1	0	0	0	1	1	1	1	5
Wu et al. [39]	1	1	0	1	0	0	0	1	1	1	1	6
Zubac and Simunic [71]	1	1	0	1	0	0	0	0	1	1	1	5

## Appendix C

See Fig. 12.
